# A blend of chitosan-vitamin C and vitamin E nanoparticles robust the immunosuppressed- status in Nile tilapia treated with salt

**DOI:** 10.1186/s12917-024-04180-y

**Published:** 2024-07-22

**Authors:** Mahmoud A. Elnagar, Riad H. Khalil, Talaat S. Talaat, Ahmed H. Sherif

**Affiliations:** 1 Fish Diseases Department, Animal Health, Research Institute (AHRI), Agriculture Research Centre (ARC), Kafrelsheikh, Egypt; 2 Fish Diseases Department, Faculty of Veterinary Medicine, Alexandria University, Alexandria, Egypt

**Keywords:** Nile tilapia, Nanoparticles, Chitosan, Vitamin C, Vitamin E, Cytokine, Antioxidant enzyme

## Abstract

In aquaculture, fish are exposed to many stressors, such as climate changes and infectious diseases that affect their performance, immunity, and welfare. Freshwater fish subjected to salt bath become exhausted and stressed. In this experiment, Nile tilapia were exposed to a salt bath at a dose of 30 ppt for 30 min a day. Vitamin C and vitamin E are well-known antioxidants that are used in aquaculture. Fish received dietary nanoparticles of chitosan-vitamin C and chitosan-vitamin E (CCE-NPs) for different periods (7 and 14 days) pre- (G2) and post-salt treatment (G3). In the control fish (G1), cortisol 5.44 µg/dL and glucose 91.67 mg/dL were significantly up-regulated post-salt treatment by 1 h and 24 h, respectively, whereas those (G2) fed CCE-NPs diet had significantly lower values of 4.72 and 3.25 µg/dL; 86.3 and 84.3 mg/dL, respectively. A rapid decrease of glucose 68.3 and 66.3 mg/dL was noticed in those (G2) fed CCE-NPs diet compared to the control 84.67 mg/dL at 48 h post-stress. Regardless of the supplementation period, fish (G2) could partially restore normal food reflex at 48 h (post-salt bath) and fully restored at 72 h compared to 7 days in the control (G1). After 48 h, fish that received dietary CCE-NPs (G2 and G3) restored normal mucus lysozyme levels, whereas the control did not restore pre-treatment values till the seventh day. Mucus antibacterial activity, fish received rapid dietary CCE-NPs (G2) and partially restored average values (pre-salt bath) at 96 h. The salt treatment could provoke gene expression of pro-inflammatory cytokines interleukin *(IL-1β)* and tumor necrosis *(TNF)-α* in the head kidney of fish at 24 h post-salt bath to 5.9–8.35 fold-change, respectively, with a rapid decline in fish (G2) the gene expression. Post-salt bath (24 h), the gene expression of glutathione peroxidase *(GPx)*, superoxide dismutase *(SOD)*, and catalase *(CAT)* was higher in fish (G2) than in the control group (G1) regardless of the supplementation period (7 and 14 days). Bacterial infection *S. agalactiae* (OL471408), a significantly lower MR was recorded in G2 at 40% and 33.3% compared to the control G1 MR (53.3%), with an RPL of 24.95% and 37.5%. In conclusion, Nile tilapia treated with a 30 ppt salt became more vulnerable to *S. agalactiae.* Adding CCE-NPs to the Nile tilapia diet for 7- and 14-day pre-salt bath could increase immune and antioxidant-related gene expression to counteract *S. agalactiae* infection.

**Supplementary Information:**

The online version contains supplementary material available at 10.1186/s12917-024-04180-y.

## Introduction

Nile tilapia *(Oreochromis niloticus )*is considered a highly cultivated freshwater fish species worldwide, becoming one of the most marketable aquatic animals. Globally, the Egyptian aquaculture sector has been ranked among the highest producers [1]. According to the latest FAO report, Egypt was named 11th among the highest aquaculture producers, with 1.591 million tons forming 1.92% and 67% of world and African production (2020), respectively [2]. In freshwater fish species, salt treatment is usually used to combat several parasitic pathogens from protozoans to helminths for its properties such as being less toxic and inexpensive compared to frequently used anti-parasitic treatments (formalin or malachite green), so it is the recommended therapy in fish [3, 4]. Meanwhile, some withdrawals from salt treatment were observed, such as impacting osmoregulation, causing dehydration [5], and developing immunosuppression status, which was controlled by the duration and dose of salt treatment [6, 7]. Salt treatment significantly disturbed the gene expression of immune-related genes, which control inflammatory responses [8], and antioxidant-related genes, which play an essential role in the detoxification of relative oxygen species (ROS) [9].

Chitosan is a natural polymer obtained from the crustacean exoskeleton. Its properties include immune-stimulant activity, biodegradability, and biocompatibility, making it an attractive candidate for functional fish feed as a coat for medicinal agents and encapsulate vaccines [10]. Moreover, chitosan nanoparticles have higher bioavailability in the bloodstream, making them more assimilated and absorbed with lesser concentration [11]. Vitamin C, an essential micronutrient, offers numerous benefits to animal health. Fish cannot biosynthesize Vitamin C, leading to its inclusion in fish feed. However, this vitamin is unstable in high temperatures, oxygen, and light [12]. Vitamin C’s antioxidant properties shield animal cells from relative oxygen species (ROS), enhancing general health, immune-stimulant, antiaging, and antimicrobial activity [13]. Vitamin E exerts an antioxidant activity that could protect fish cells and tissues from injuries caused by ROS generated in stressful circumstances [14]. Studies confirmed the immune-stimulant effects of vitamin E (essential fat-soluble micronutrient) in fish [14]. “Alpha-tocopherol” is one of the eight forms of vitamin E commonly added to fish feed for its significant health impacts. Also, they added that vitamin E could improve both innate and required immunity, decreasing mortality rate and improving growth performance. It could also protect the functions of fish leukocytes [15].

Our recent breakthrough involves the development of a novel nanotechnology-based drug formula. This cutting-edge technology has been approved as an alternative to traditional drug manufacturing methods, offering biocompatible nanocomposites such as nanocapsules, nanoparticles, and conjugates [16]. These innovative techniques protect unstable environmental materials like vitamin C, reduce waste, and preserve physicochemical properties [12].

This work assessed immune-antioxidant responses and physiological status in Nile tilapia treated with salt. A trial was conducted to mitigate the stress associated with salt treatment using a dietary blend of nano-sized chitosan capsulated vitamin C and vitamin E (CCE-NPs). Also, the ability of fish that received dietary CCE-NPs with different schedules to counteract *Streptococcus agalactaie* infection.

## Materials and methods

### Fish accommodation, experiment design, nanoparticles preparation

A two hundred and seventy Nile tilapia *(O. niloticus)* were purchased from local fish farms in Kafrelsheikh Governorate, weighing 50 ± 1.3 g. Prior to the transportation, fish were tranquilized using 40 mg/L tricaine methanesulfonate (MS-222, Syndel, Canada) in the fish farms and then rapidly transported in the containers supplied with aerated water. At the wet laboratory, Nile tilapia were subjected to an iodine bath of Betadine^®^, the active ingredient 5% of povidone-iodine, and produced by the Nile Company for Pharmaceuticals [17, 18]. In A glass tank (3 × 1.5 × 1 m), fish were stocked for 14 days of acclimatization, and fish were fed a commercial diet (mentioned below) once daily at 09:00 am. After that, fish were randomly distributed and stocked in glass aquaria (50 × 40 × 40 cm) containing about 60 L. The Tank and aquaria contained dechlorinated tap water, supplied with oxygen via air stone with an electric compressor; the water quality was maintained to be suitable for optimal fish culture by replacing one-third of the water with clean, fresh, and dechlorinated water day after day to avoid wastes accumulation. The water parameters were suitable for the fish culture temperature (28 ± 0.5 °C), salinity (0.15 ± 0.03 g/L), hydrogen ion pH (7.4 ± 0.1), and dissolved oxygen DO (5.4 ± 0.3 mg/L), total ammonia nitrogen TAN (0.08 ± 0.02 mg/L), unionized ammonia NH_3_ (0.01–0.00 mg/L), nitrite NO_2_ (0.00 mg/L), and nitrate NO_3_ (0.4 ± 0.02 mg/L).

In the experimental design, fish were exposed to a salt bath three times (once per day) with a day interval [19] by adding 30 g salt/L (30 ppt) for 30 min, and the feeding process stopped during treatment. Feeding a nanoparticles blend (CCE-NPs) of chitosan-vitamin C (Ch-CNPs) and chitosan-vitamin E (Ch-ENPs) was done using the optimal dietary level of vitamin C and E for Nile tilapia 420 and 100 mg/kg dry diet, respectively, following the recommendations of **NRC** [20].

Fish was distributed into three groups, G1–3, each with three replicates (30 fish/aquaria). Fish of G1 served as the control; they were not subjected to a salt bath or received feed additives. Fish of G2 were fed dietary CCE-NPs for 7 and 14 days before being subjected to the salt bath. Fish of G3 were fed dietary CCE-NPs for 7 and 14 days after being subjected to the salt bath.

The sampling procedure was performed before and after the salt bath and 7 and 14 days post-salt bath. Fish were euthanized using MS-222 at a dose of 250 mg/L, and they were kept for ten minutes after ceasing the operculum movements [19].

Chitosan nanoparticle preparation (ionotropic-gelation method) was synthesized depending on electrostatic interaction between charged negative and positive molecules. Amino groups of chitosan interact with tripolyphosphate with negatively charged groups. Chitosan solution was made at 1.5 g dissolved in 300 ml of acidified distilled water with 3 ml Glacial acetic acid by vigorous stirring until a transparent solution was observed. The solution pH was up to 4.5 using NaOH, then filtered to remove all undissolved materials. Vitamin C 420 mg/vitamin E 100 mg was mixed with TPP (100 mg/100 ml DW) and added drop-wise at a consistent rate using a titration pipette at the rate of 1 ml/minute under continuous stirring at room temperature for 2 h then the mixture was sonicated for 10 min [21]. The solution was centrifuged at 14,000 rpm at four °C/30 min twice with washing, and the supernatant was discarded. The resulting sediment dissolved in distilled water and well-ground, then lyophilized for further investigation. The nanoparticle characterizations were performed using high-resolution transmission electron microscopy (JEM1400F HRTEM equipped with a 300 keV beam energy) at the Faculty of Agriculture, Cairo University.

Preparation of fish feed: firstly, the consistency and viscoelastic of the commercial fish feed pellets were done by soaking them in water and blended to form a paste, nanoparticles were added to the food past and thoroughly mixed with gelatin (Nutri-B-Gel) produced by Canal Aqua Cure (Port-Said, Egypt) 5% w/w to enhance consistency. Fish feed was allowed with 5% fish body weight twice daily at 09:00 am and 0.3:00 pm. Fish feed composition was as follows: Moisture 11.1%, Crude Protein 42.72%, Digestible Energy 2955.62 (Kcal/Kg), Ether extract 5.74%, Crude fiber 2.6%, Nitrogen free extract 35.3%, and Ash 7.4%)

### Chemicals used in the experiment

All chemicals were purchased from the local market, and Sigma-Aldrich produced salt. Chitosan was produced by Sigma-Aldrich, USA, with a low molecular weight (MW) of 50–90 kDA and a de-acetylation degree of ≥ 75% based on viscosity. Vitamin C Catalog codes: SLA1306 SLA4315 (Sciencelab.com et al., Texas, USA). Vitamin E, GV1022DL-alpha-Tocopherol acetate, EP/USP/FCC GRAD CAS RN 7695-91-2 Glentham Life Science England.

### Stress hormone and glucose

Serum level of cortisol, a widely used stress bioindicator, was measured at 0, 1, 6, 12, 24, and 48 h post-salt treatment [22, 23] using ELISA at an absorbance of 415 nm, 96-well, kits (Cayman Chemical, USA), and Microplate Reader (Azure Biosystems, USA). This measurement is crucial in understanding the physiological response to stress in the fish species under study. Serum samples were collected at 0, 1, 6, 12, 24, 48, 72, and 96 h post-salt treatment, and glucose levels were measured using a Spinreact^®^ glucose test kit.

### Fish food reflex

At 0, 1, 6, 12, 24, 48, 72, 96 h, 7 days, and 14 days post-salt treatment, Nile tilapia feed was offered, and time of the food approach was calculated [24], scores were 1–4 based on finishing the offered food, 1: in ≤ 120 s, 2: in 120–180 s, 3: in 180–300 s, and 4: No or ≥ 360 s.

### Noninvasive analyses of the mucus

Skin mucus was collected from five fish per group at 0, 1, 6, 12, 24, 48, 72, 96 h, 7 days, and 14 days post-salt treatment. For 30 s, each fish was gently rubbed in a plastic bag containing 10 mL of 50 mM NaCl, then centrifuged at 1500 rpm at 4 °C for 10 min and preserved at -96 °C.

The lysozyme level in skin mucus was quantified using the Enzyme-Linked Immunosorbent Assay (ELISA) method, a widely accepted technique for its sensitivity and specificity described by **Parry et al.** [25].

Skin mucus antibacterial activity was measured following the method described by **Kumari et al.** [26]. A blend of mucus (100 µL) and saline (6.5 g/L) was vortex-mixed in triplicate with *Aeromonas hydrophila* AHRAS2 (accession numbers of MW092007 in GenBank that isolated by **Sherif and Abuleila**, [27]**).** The bacterial suspension containing 10^6^ CFU was incubated at 25 °C/1 h. Then, the mix was incubated at 25 °C/24 h. The antibacterial activity was determined as a percentage of live colonies to the primary bacterial number.

### Gene expression of immune and antioxidant-related genes

The impact of salt treatment on the expression of immune-related genes (Table 1) was assessed using quantitative real-time polymerase chain reaction (RT-PCR). The RNA was extracted from the head kidney with Trizol reagent (iNtRON Biotechnology Inc., Korea), and samples were collected from three Nile tilapia, each group using Nanodrop D-1000 spectrophotometer (NanoDrop Technologies Inc., USA). The obtained RNA was assessed for quality and quantity and kept at − 80 °C. The β-actin was the housekeeping gene. The results were assessed using Eq. 2^−ΔΔCT^ [28].

**Table 1:** List of all primers

Target gene	Primer sequence	Amplified segment Length	Annealing temperature	Accession number
*β-actin*	F: GCATCACACCTTCTACAACGA R: TGGCGGGGGTGTTGAAGGTCT	139 bp	57 °C 30 s	AA566386
*IL-1β*	F: T GCTGAGCACAGAATTCCAG R: GCTGTGGAGAAGAACCAAGC	172 bp	60 °C 30 s	XM_019365841.2
*TNF-α*	F: CCAGAAGCACTAAAGGCGAAGA R: CCTTGGCTTTGCTGCTGATC	82 bp	59.9 °C 30 s	AY428948.1
*IL-10*	F: CTGCTAGATCAGTCCGTCGAA R: GCAGAACCGTGTCCAGGTAA	94 bp	60 °C 30 s	XM_013269189.3
*GPx*	F: CCAAGAGAACTGCAAGAACGA R: CAGGACACGTCATTCCTACAC	237	58 °C 30 s	NM_001279711.1
*SOD*	F: GGTGCCCTGGAGCCCTA R: ATGCGAAGTCTTCCACTGTC	377	56 °C 30 s	JF801727.1
*CAT*	F: TCCTGAATGAGGAGGAGCGA R: ATCTTAGATGAGGCGGTGATG	232	56 °C 30 s	JF801726.1

Note: *β-actin;* housekeeping gene, *IL-1β;* Interleukin-1 beta, *TNF- α;* tumour necrosis factor alpha, *GPx;* glutathione peroxidase, *SOD;* superoxide dismutase, and *CAT;* catalase

### Bacterial infection

At 0, 1, 7, and 14 days, post-salt treatment, ten Nile tilapia per group were randomly and intraperitoneally (IP) injected with *S. agalactiae* with NCBI accession number (OL471408). Its median lethal dose (LD_50_) is 0.3 × 10^5^ CFU/ml, previously isolated and identified by **Sherif et al.** [29]. In addition, ten fish from the control group were injected with pure saline 6.5 g/L as negative controls. The injected Nile tilapia were observed for fourteen days to record the fish deaths. The mortality rate (MR %) and the relative levels of protection (RLP) of CCE-NPs were calculated according to the following equations:

$$\:\text{M}\text{R}\left(\text{\%}\right)\:=\frac{\text{n}\text{u}\text{m}\text{b}\text{e}\text{r}\:\text{o}\text{f}\:\text{d}\text{e}\text{a}\text{t}\text{h}\text{s}\:\text{i}\text{n}\:\text{a}\:\text{s}\text{p}\text{e}\text{c}\text{i}\text{f}\text{i}\text{c}\:\text{p}\text{e}\text{r}\text{i}\text{o}\text{d}\:}{\text{t}\text{o}\text{t}\text{a}\text{l}\:\text{p}\text{o}\text{p}\text{u}\text{l}\text{a}\text{t}\text{i}\text{o}\text{n}\:\text{d}\text{u}\text{r}\text{i}\text{n}\text{g}\:\text{t}\text{h}\text{a}\text{t}\:\text{p}\text{e}\text{r}\text{i}\text{o}\text{d}}\times\:\:100$$

$$\:\text{R}\text{L}\text{P}\text{\%}=(1-\frac{\text{\%}\text{d}\text{e}\text{a}\text{t}\text{h}\text{s}\:\text{i}\text{n}\:\text{t}\text{h}\text{e}\:\text{t}\text{r}\text{e}\text{a}\text{t}\text{e}\text{d}\:\text{g}\text{r}\text{o}\text{u}\text{p}}{\text{\%}\text{d}\text{e}\text{a}\text{t}\text{h}\text{s}\:\text{i}\text{n}\:\text{t}\text{h}\text{e}\:\text{c}\text{o}\text{n}\text{t}\text{r}\text{o}\text{l}\:\text{g}\text{r}\text{o}\text{u}\text{p}})\times\:\:100$$

### Statistical analyses

The effects of nano-vitamins CCE-NPs modulation of Nile tilapia immune-antioxidant status using two-way ANOVA and Duncan’s Multiple Range at significant *P* values less than 0.05 using SPSS version 2022 software. Data presented as mean ± standard error (SD).

## Results

### Nano material

**Fig. 1:**
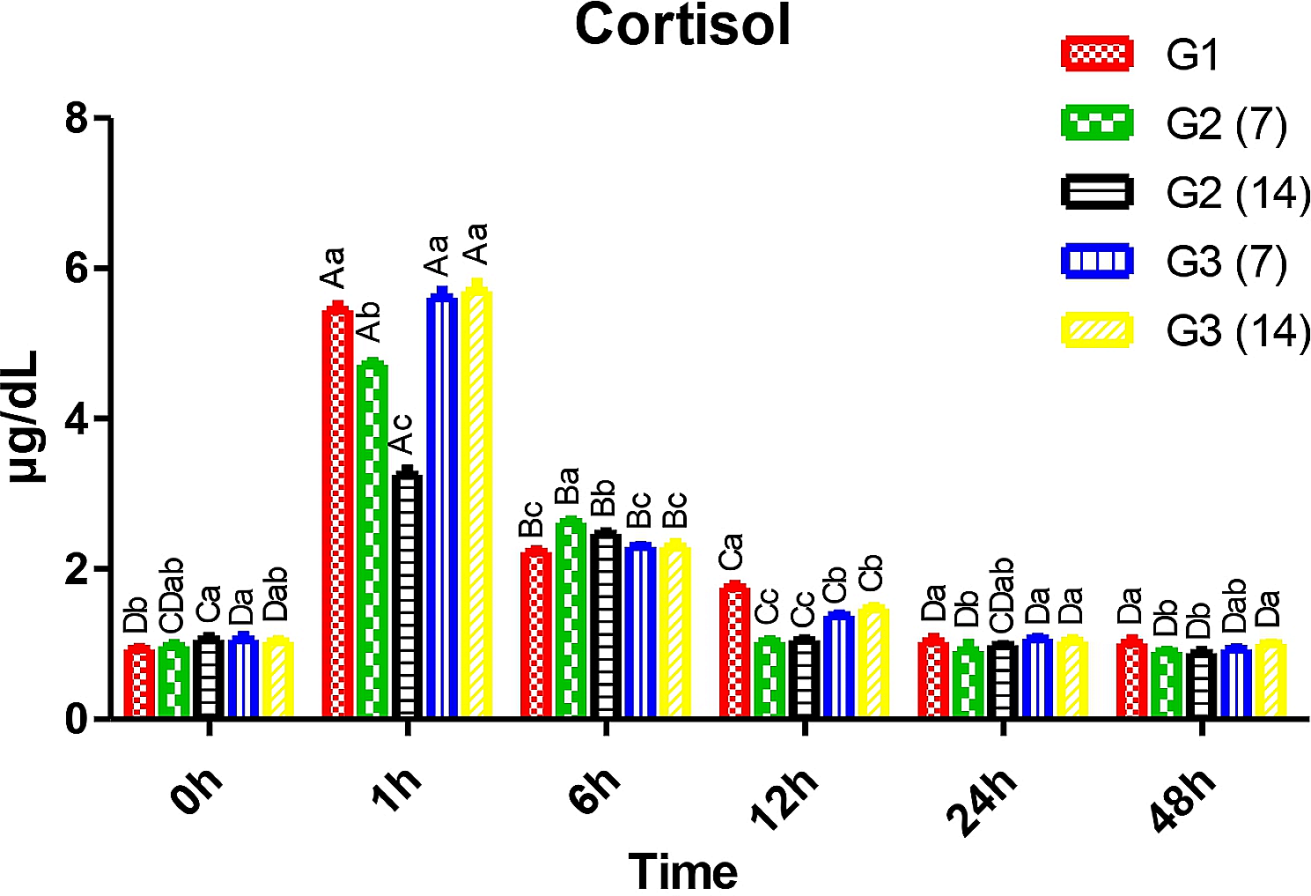
Cortisol level. Different capital letters (time factor) and small letters (additive factor) indicate that values are significant differences at *P* ≤ 0.05. G1; Control without stress or feed additives, G2; Fish fed CCE-NPs pre-salt treatment 7 and 14 days, G3; Fish fed dietary (CCE-NPs) post-salt treatment 7 and 14 days

**Fig. 2:**
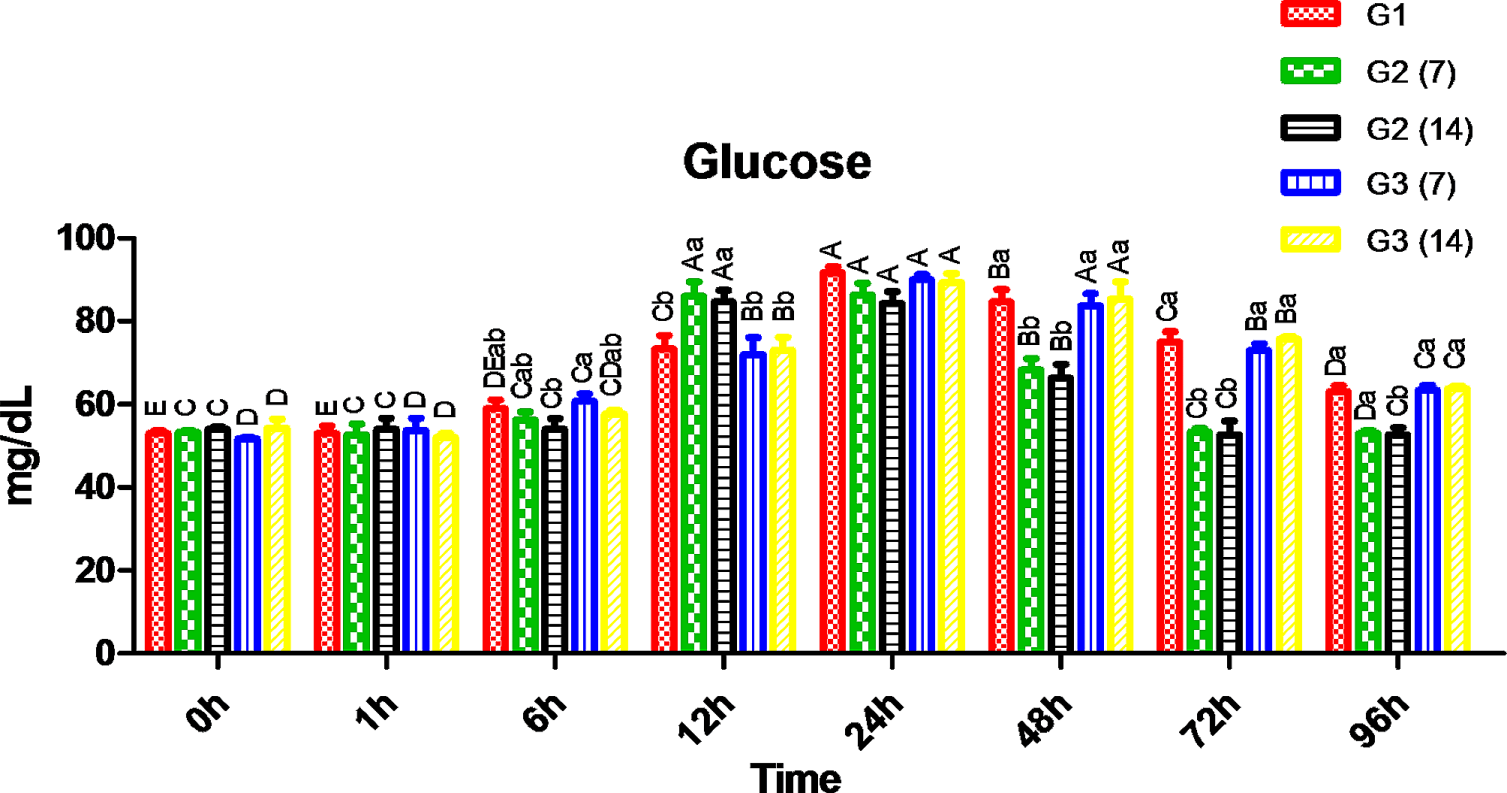
Serum glucose level. Different capital letters (time factor) and small letters (additive factor) indicate that values are significant differences at *P* ≤ 0.05. G1; Control without stress or feed additives, G2; Fish fed CCE-NPs pre-salt treatment 7 and 14 days, G3; Fish fed dietary (CCE-NPs) post-salt treatment 7 and 14 days

In this experiment, nano-composite (CCE-NPs) was composed of chitosan-vitamin C and chitosan-vitamin E, with sizes ranging from 11.8 to 14.1 nm and 16.3 to 23.3 nm, respectively. Each gram of (CCE-NPs) contained 420 mg of vitamin C and 100 mg of vitamin E (Supplementary; Figs. 1 and 2).

### Stress indicators

In Fig. 1, the salt bath was stressful for the experimental fish and was assessed by measuring serum cortisol (stress hormone) at 0, 1, 6, 12, 24, and 48 h, while serum glucose was measured at 0, 1, 6, 12, 24, 48, 72, and 96 h. Pre-salt treatment (0 h) cortisol ranged between 0.93 and 1.06 µg/dL. After 1 h, Nile tilapia (G2) supplemented with CCE-NPs for 7 and 14 days had significantly lower levels of 4.72 and 3.25 µg/dL, respectively, and serum levels declined rapidly compared to the other groups.

In Fig. 2, after 12 h, fish fed with CCE-NPs (G2) for 7 and 14 days and then exposed to salt treatment had significantly higher serum glucose levels, 86.3 and 84.3 mg/dL, which started to decline after 48 h and restored normal values after 72 h compared to the pre-salt bath (0 h). Meanwhile, other groups had significantly higher levels, 89.3 to 91.67 mg/dL, after 24 h compared to the pre-salt bath (0 h), and normality was restored after 96 h.

Food reflex is the first noticed clinical sign in stressed fish. All experimental fish stopped feeding with lethargy swimming in the first 12 h after salt-treatment exposure. After 24 h, fish received dietary CCE-NPs (G2) rapidly and partially restored normal foods reflex (4) and enhanced in 48 h (post-stress). Then, at 72 h, they had fully restored normality; meanwhile, the other groups did not restore till day 7 (Table 2).

**Table 2:** Fish food reflex

Items	G1 (control)	G2 (7 days)	G2 (14 days)	G2 (7 days)	G3 (14 days)
Pre-stress(0 h)	1	1	1	1	1
1 h	-	-	-	-	-
6 h	-	-	-	-	-
12 h	-	-	-	-	-
24 h	-	4	4	-	-
48 h	4	3	3	4	4
72 h	3	1	1	3	3
96 h	3	1	1	2	2
7 days	2	1	1	1	1
14 days	1	1	1	1	1

Note: G1; Control without stress or feed additives, G2; Fish fed CCE-NPs pre-salt treatment 7 and 14 days, G3; Fish fed dietary (CCE-NPs) post-salt treatment 7 and 14 days. Scores were 1–4 based on finishing the offered food, 1: in ≤ 120 s, 2: in 120–180 s, 3: in 180–300 s, and 4: No or ≥ 360 s

**Fig. 3:**
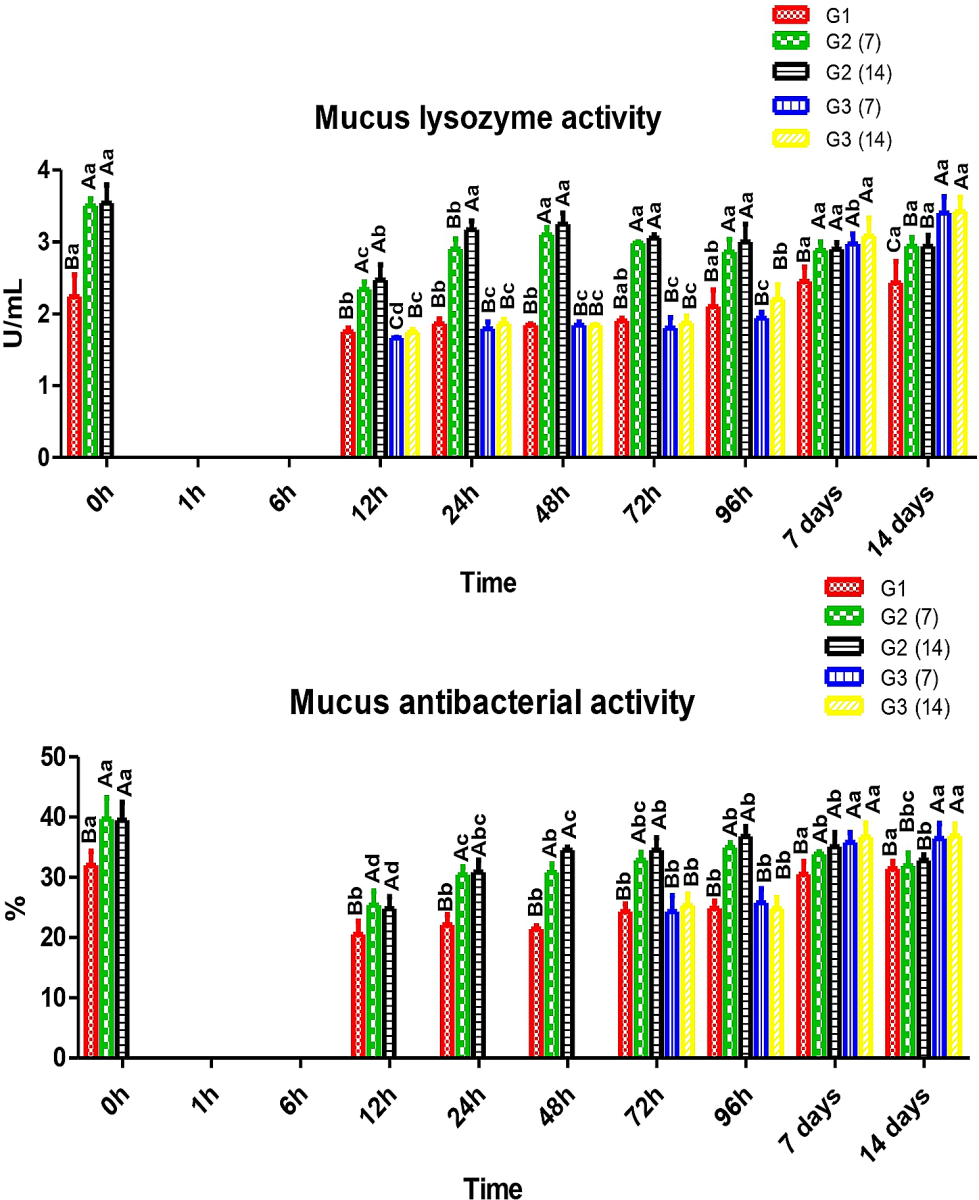
Mucus lysozyme and antibacterial activity. Different capital letters (additive factor) and small letters (time factor) indicate that values are significant differences at *P* ≤ 0.05. G1; Control without stress or feed additives, G2; Fish fed CCE-NPs pre-salt treatment 7 and 14 days, G3; Fish fed dietary (CCE-NPs) post-salt treatment 7 and 14 days

In Fig. 3, Nile tilapia was treated every other day with a salt bath. Mucus was scarce to be collected during the first 6 h after the salt bath. After 12 h, mucus lysozyme was significantly higher in fish (G2) that received dietary CCE-NPs for 7 and 14 days, 2.34 and 2.47 U/mL, respectively, compared to other groups 166–1.75 U/mL. After 48 h, fish of (G2) restored pre-stress lysozyme levels, whereas the other group did not restore pre-stress values till 7 days. Also, fish of G2 had superiority over the other groups till 14 days post-stress.

Antibacterial activity (Fig. 3) of fish mucus was drastically impacted by exposure to salt bath. Fish received dietary CCE-NPs (G2) rapidly and partially restored normal values (pre-salt bath) to 96h, whereas it required 7 days in the other groups. Fish (G3) received dietary CCE-NPs had higher activities compared to (G2) after 7 days, 25.8% and 36.7%; 33.8% and 35.1%, also the superiority extended after 14 days, 36.45% and 36.9%; 31.9% and 32.8%, respectively.

### Cytokines gene expression

**Fig. 4:**
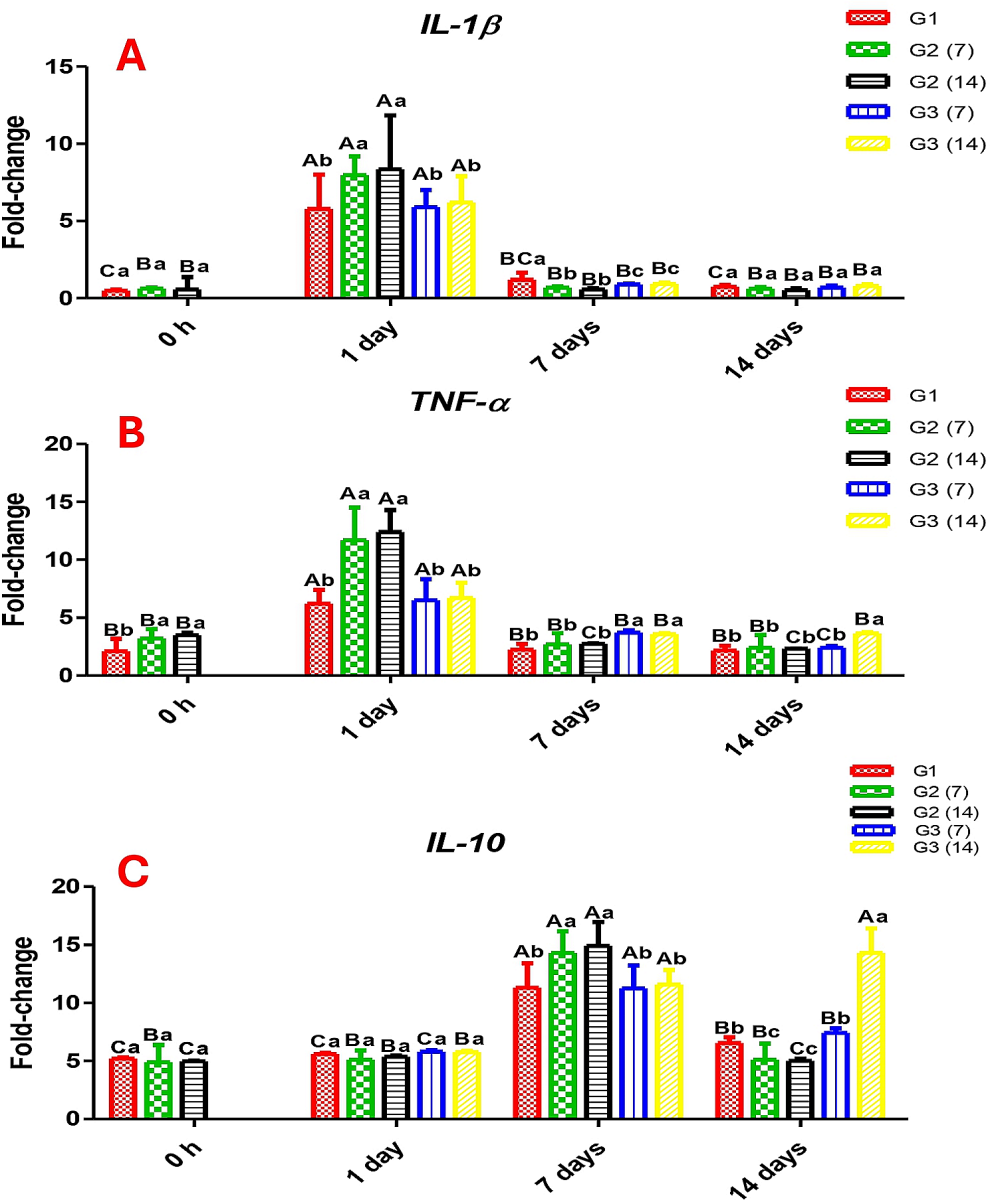
Gene expression of (**A**) interleukin *(IL)-1β*, (**B**) tumor necrosis factor *(TNF)-α*, and (**C**) interleukin *(IL)-*10. Different capital letters (time factor) and small letters (additive factor) indicate that values are significant differences at *P* ≤ 0.05. G1; Control without stress or feed additives, G2; Fish fed CCE-NPs pre-salt treatment 7 and 14 days, G3; Fish fed dietary (CCE-NPs) post-salt treatment 7 and 14 days

In Fig. 4, the gene expression of *IL-1β*,* TNF-α*,* and IL-10* was modulated in the head kidney, indicating that salt treatment could provoke gene expression of cytokines. After 24 h of salt bath, *IL-1β* and *TNF-α* showed significantly higher expressions than the control regardless of the period of CCE-NPs supplementation and declined on day 7. The expression of *IL-10* was increased on day 7 after the salt bath regardless of the group. At 14 days post-salt bath, the expression of *IL-10* in fish of G3 was significantly higher than in the other groups.

### Antioxidants gene expression

**Fig. 5:**
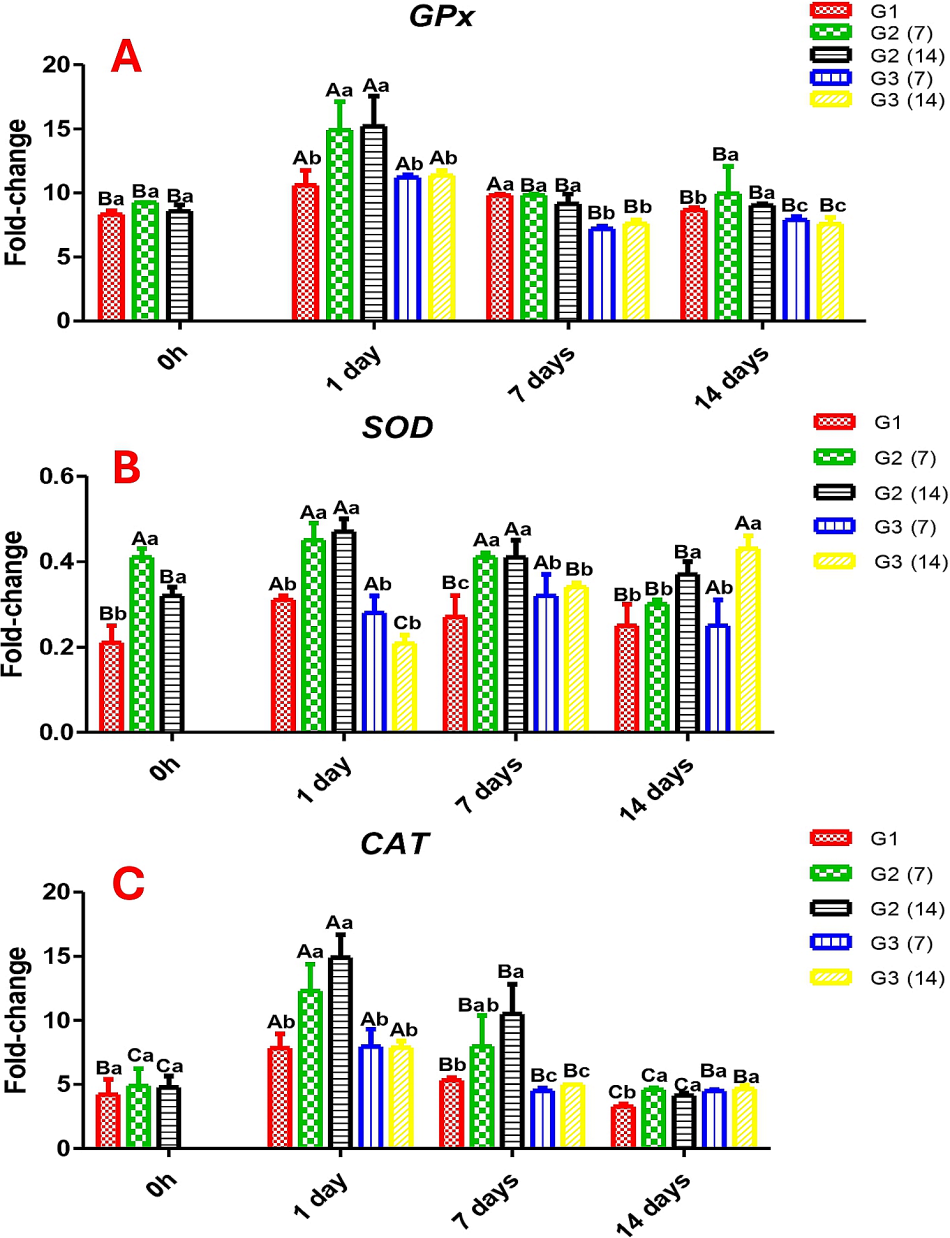
Gene expression of (**A**) glutathione peroxidase *GPx*, (**B**) Superoxide dismutase *SOD*, and (**C**) Catalase *CAT.* Different capital letters (time factor) and small letters (additive factor) indicate that values are significant differences at *P* ≤ 0.05. G1; Control without stress or feed additives, G2; Fish fed CCE-NPs pre-salt treatment 7 and 14 days, G3; Fish fed dietary (CCE-NPs) post-salt treatment 7 and 14 days

In Fig. (5), in the head kidney, the gene expression of antioxidant *GPx* was significantly higher in fish of G2 (CCE-NPs supplementation) than in the control group (G1) regardless of the supplementation period (7 and 14 days), 9.24 and 8.57 fold-change, respectively. In addition, *SOD* and *CAT* had the same trend of *GPx* (Fig. 5).

Post-stress (1 day) (Fig. 5), the gene expression of *GPx* and *CAT* raised in G2 was significantly higher than in the pre-stressed condition (0 h) and the other stressed groups. Meanwhile, the expression of *SOD* did not affect by salt treatment.

Post-stress, all groups possessed normal values, but fish supplemented with CCE-NPs were still high regardless of the period of addition (7 and 14 days) and time (pre- or post-stress) (Fig. 5).

### Bacterial infection

The dietary CCE-NPs showed promising results. They provided RPL of 24.95% and 37.5% for Nile tilapia in (G2), which fed dietary CCE-NPs for 7 or 14 days pre-treatment, respectively, against experimental infection with LD_50_ of *S. agalactiae.* At 24 h- post-salt treatment, RPL increased to 42.87% regardless of the supplementation period. High RPL demonstrates the potential of our dietary intervention to improve fish health. At 7 days post-infection, RPL decreased to 11.6 and 0%, respectively, whereas at 14 days post-infection, RPL reached 10% for both periods (G2). Meanwhile, RPL increased in (G3), which fed dietary CCE-NPs for 7 or 14 days post-salt bath, to 44.5% and 44.5% at 7 days post-infection and 20.05% and 50.5% at14 days post-infection, respectively, (Table 3).

**Table 3:** Nile tilapia mortality rate and relative levels protection after experimental infection with *S. Agalactiae* (LD_50_)

Items		Control (-ve)	G1 (control + ve)	G2 (7 days)	G2 (14 days)	G2 (7 days)	G3 (14 days)
0 h	MR no. (%)	2 **(13.3)**	8 **(53.3)**	6 **(40)**	5 **(33.3)**	7 **(46.67)**	8 **(53.3)**
	RPL (%)	-	-	24.95	37.5	-	-
24 h	MR no. (%)	2 **(13.3)**	14 **(93.3)**	8 **(53.3)**	8 **(53.3)**	13 **(86.67)**	14 **(93.3)**
	RPL (%)	-	-	42.87	42.87	-	-
7 days	MR no. (%)	2 **(13.3)**	9 **(60)**	8 **(53.3)**	9 **(60)**	5**(33.3)**	5 **(33.3)**
	RPL (%)	-	-	11.16	0	44.5	44.5
14 days	MR no. (%)	2 **(13.3)**	10 **(66.67)**	9 **(60)**	9 **(60)**	8**(53.3)**	5 **(33.3)**
	RPL (%)	-	-	10.0	10.0	20.05	50.5

Note: (*n* = 15), Control (-ve); control fish injected with saline, G1; Control without stress or feed additives, G2; Fish fed CCE-NPs pre-salt treatment 7 and 14 days, G3; Fish fed dietary (CCE-NPs) post-salt treatment 7 and 14 days

**Fig. 6:**
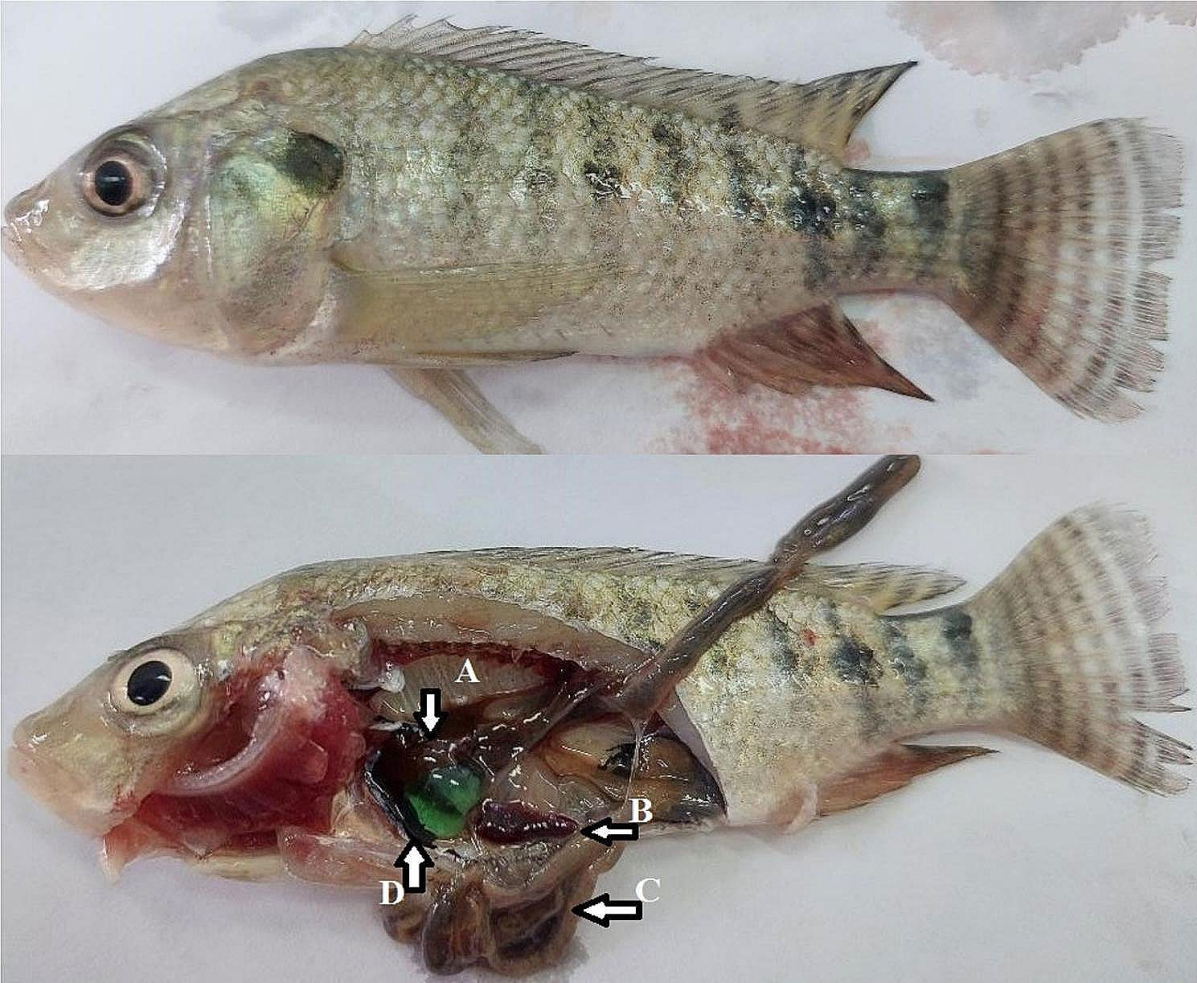
Nile tilapia (control), clinical sign yellowish discoloration with large head and tail fin, postmortem (**A**) dark brownish-reddish liver, (**B**) splenomegaly (**C**) partial empty intestine, (**D**) distended gall-bladder with clear content

**Fig. 7:**
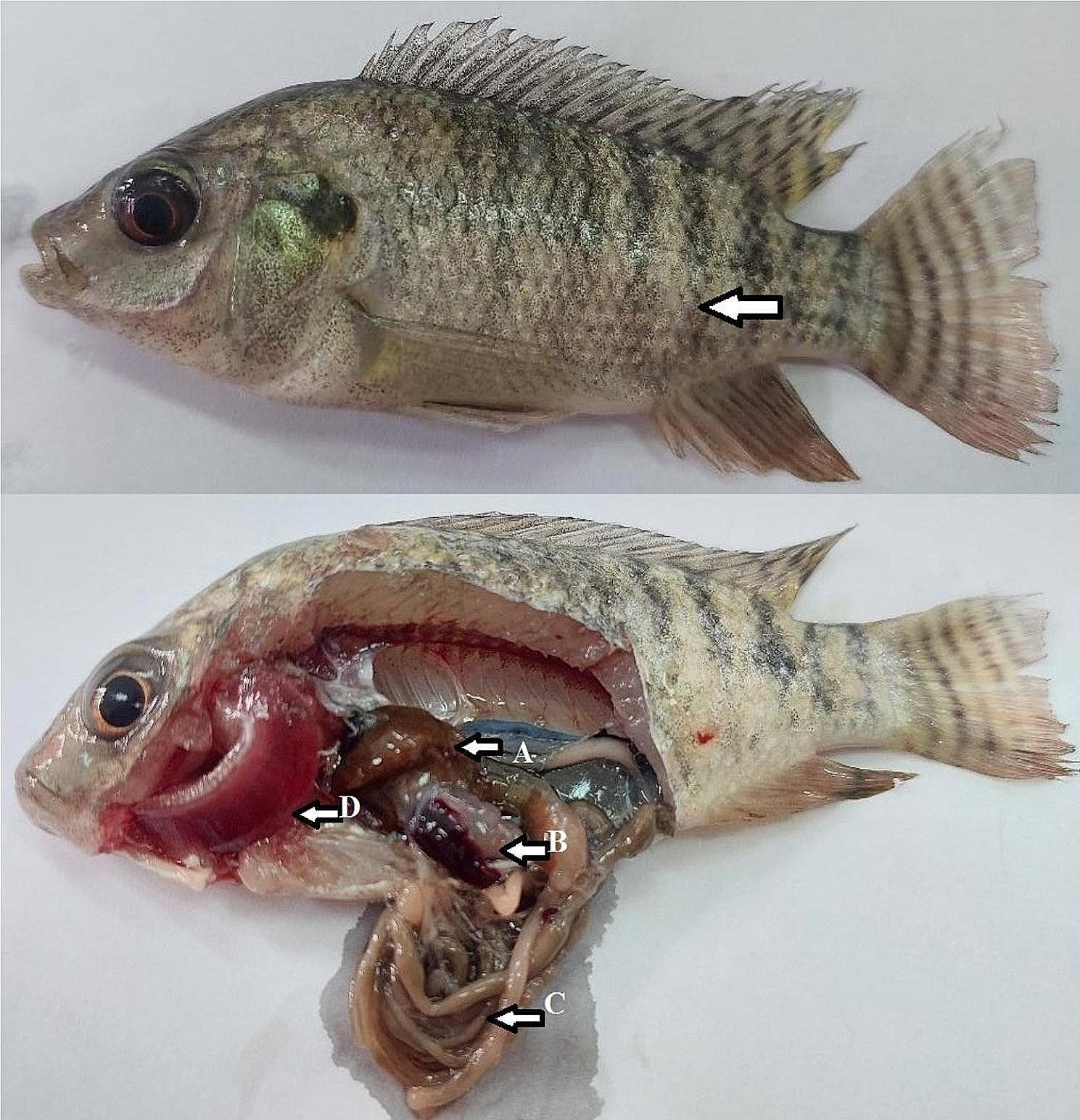
Nile tilapia treated with salt and fed dietary-CCE-NPs, clinical sign with external slight yellowish discoloration, postmortem (**A**) brownish liver, (**B**) splenomegaly, (**C**) full-intestine, and (**D**) pale gills

After exposure to salt treatment (Fig. 6), Nile tilapia experimentally infected with *S. agalactiae* had signs of off–food (large head and tail fin) with yellowish discoloration of the skin, partial empty intestine, and distended gall-bladder with clear content. The post-mortem showed bacterial septicemic signs: dark brownish-reddish liver and splenomegaly. After exposure to salt treatment (Fig. 7), Nile tilapia infected with *S. agalactiae* (LD_50_) and supplemented with dietary CCE-NPs had clinical signs that were similar to the unsupplemented group with slight intensity, slight yellowish discoloration of the skin, partial empty intestine, in addition the post-mortem lesions were brownish liver, splenomegaly, and pale gills.

## Discussion

Our research findings highlight the significant role of CCE-NPs in mitigating the stress in Nile tilapia caused by salt treatment. Serum cortisol and glucose, stress-related parameters, are crucial indicators of stress conditions in fish [30]. In our study, Nile tilapia that received dietary-CCE-NPs for 7 and 14 days pre-salt bath had significantly low cortisol levels in serum (4.72 and 3.25 µg/dL) compared to the control fish (5.44 µg/dL). The high cortisol levels were reported in the serum of common carp reared in high salinity concentrations [31]. Similarly, **Karsi and Yildiz** [32] observed similar findings in Nile tilapia after direct transport to salt water (9 and 18 ppt) for 72 h. These results further confirm the vital role of vitamin E in regulating plasma cortisol concentration under stress [33].

During stress, hyperglycemia levels are controlled with stress types and sampling times [34]. In this experiment, salt treatment caused high glucose levels in the serum of Nile tilapia. The glucose levels were significantly higher, 86.3 and 84.3 mg/dL, after 12 h in the serum of fish that received dietary CCE-NPs pre-salt bath for 7 and 14 days, respectively. Also, they restored the basal level after 72 h of salt bath, compared to the control fish that needed more than 96 h. Accordingly, high glucose levels are released under cortisol control during stress to afford the entire body the energy required to counteract the stress, glucose levels were significantly decreased in the serum of stressed Nile tilapia received a dietary vitamin C [35].

In this work, even though the control fish could withstand salt stress, some fish showed a low food reflex, which was the first clinical sign of stress. Also, skin mucus was scarce and could not be collected in the first 6 h post-stress, which drastically impacted the antibacterial activity of the mucus. Salt treatment changes the osmotic hemostasis of fish parasites and strips off the protective mucus layer, depriving the parasite of protection against an adverse environment [36]. It is known that environmental stress causes an increase in mucus production, resulting in the depletion of mucus cells. The linear increase of mucus production in response to salt stress until depletion is slow at 3 ppt compared to the 7 ppt group [37].

Dietary CCE-NPs (pre-stress 7 and 14 days) resulted in significantly higher mucus lysozyme 2.34 and 2.47 U/mL at 12 h post-stress in experimental Nile tilapia, rapidly restoring basal level after 48 h. In contrast, the other experimental groups spent 7 days restoring pre-stress value. Similarly, **Alishahi et al.** [38] illuminated that the highest lysozyme activity was recorded in rainbow trout fed on a diet supplemented with nano-chitosan loaded with vitamin C and silver carp. Higher lysozyme activity in rainbow trout fed a diet containing vitamin C + vitamin E + Nano-Se, while Ig and ACH50 insignificantly differ among the experimental groups [39].

In accordance, **Tort** [6] reported that the extent of immune depression status was associated with stressors kinds, such as acute (short-term) and chronic (long-term), that controlled the probability of infection. In this study, 24 h post-salt bath, the expression of *IL-1β* and *TNF-α* genes in the head kidney of the experimental Nile tilapia was increased, regardless of the CCE-NPs supplementation period, with a rapid decline on day 7, in parallel with the increase of the expression of *IL-10.* Similarly, **El-Leithy et al.** [40] found that the expression of *IL-1β*,* IL-8*, and cc-chemokine genes in the liver of Nile tilapia was significantly higher when exposed to salt 16 ppt than with 20 ppt. Accordingly, the expression of the *IL-1β* gene was significantly increased in zebrafish larvae that received dietary chitosan NPs [41]. Moreover, Nile tilapia supplemented with dietary vitamin C had high expression levels of *TNF-α* and *IL-1β* genes [42]. Along with our results, incorporating dietary nano-Se and vitamin C or E is comparable to immunostimulants that could efficiently regulate pro-inflammatory cytokines *TNF-α* and *IL-1β* [43]. Furthermore, it was noted that dietary immunostimulants significantly increased the transcription of *IL-1β* and *TNF-α* genes in the head kidney of Nile tilapia [44–47].

The activity *GPx*,* SOD*, and *CAT*, antioxidant enzymes, could serve as biomarkers of the occurrence of oxidative stress. Also, changes in their levels are good indicators of aquatic animals’ antioxidant responses. In this experiment, Nile tilapia that were exposed to high salt concentration showed generated ROS manifested with induced high gene expression of *GPx*,* SOD*, and *CAT.* In accordance, high salinity and unstable environmental conditions are responsible for the production of free radicals (ROS) [48]. At hyper-salinity 16-ppt, the expression of glutathione gene was significantly upregulated in Nile tilapia gills to 91.1-fold change whereas at 20-ppt salt resulted in a lesser increase [40], less increase at the concentration 20 ppt could be due to fish exhaustion [49]. Conversely, the glutathione concentration was insignificantly changed in the liver of Chinook salmon at 16- and 20-ppt salt concentrations [50].

The experimental Nile tilapia that received dietary CCE-NPs for 7 and 14-day pre-salt treatment had higher gene expression of *GPx*,* SOD*, and *CAT* compared to the control, which remained high for 14 days post-stress. In accordance, it was reported that enhanced antioxidant activity by dietary incorporation of chitosan nanoparticles in Nile tilapia diets through an increase in the activity of CAT and SOD enzymes [51]. Also, CAT activity in the kidney and liver tissues was the highest in the fishes fed with the chitosan-NP at a 1 g/kg diet; this activity is attributed to chelating the metal ions and scavenging the free radicals [52]. Similarly, a study found that vitamin E supplementation significantly enhanced SOD activity in serum and muscle and CAT activity in serum, claiming that dietary vitamin E is a fast-acting antioxidant reducing the oxidative stress in large yellow croaker *(Larimichthys crocea)* [53]. Interestingly, in a 70-day feeding trial, chitosan vitamin E nanocomposite (300 mg/kg) ameliorated the high stocking density of Nile tilapia (14.74 g b.w.) and improved the serum and hepatic antioxidant enzymes [54]. In contrast, dietary E could insignificantly increase GPx activity without a synergistic effect with selenium [55]. Vitamin C is an antioxidant that protects animal cells from oxidative stress by detoxifying and neutralizing ROS [56]. It has been reported that the expressions of *GPx1a* and *gpx4b* genes were upregulated in the kidney and spleen of young grass carp (*Ctenopharyngodon idella*) by receiving dietary vitamin C at a dose of 2,9–224,5 mg/kg fish feed [57]. On the contrary, dietary vitamin C at a dose of 1.5 g/L caused significant and insignificant downregulation of the expressions of *SOD1* and *SOD2* genes, respectively, in skeletal muscle [58].

The salt bath at a concentration of 30 ppt for 30 min was stressful for the experimental Nile tilapia. A concentration of 18.9 ppt was the 96-h Median Lethal Salinity (MLS-96) for Nile tilapia fry of 7- to 120-day-old after direct transfer from freshwater [59]. Nile tilapia received dietary CCE-NPs for 7 and 14 days pre-salt bath could withstand *S. agalactiae* infection, showing low MR 40% and 33.3%, respectively. Also, it could protect Nile tilapia, providing a RPL of 24.95% and 37.5%. Whereas fish of (G3), fed dietary CCE-NPs for 7 or 14 days post-treatment, had higher RPL than the control. Accordingly, stress from high salt results in the immune depression status in fish, which makes them more vulnerable to an infectious agent present in the aquatic environment [60]. Also, high salinity is a more suitable and favorable environment for many pathogenic agents, increasing their load in water and raising the chances of infection occurrence in fish [6]. Also, it was observed that a lower concentration of chitosan-NPs would not be able to protect the fish, but the protection level was increased as the dose increased [61]. Furthermore, zebrafish larvae fed dietary-chitosan NPs could combat *A. hydrophila* infection [41], *Staphylococcus aureus* in Silver carp [62], *Vibrio alginolyticus*, *V. anguillarum*,* A. hydrophila*, and *A. veronii* [63]. Similarly, **Ahmed et al.** [53], in a 70-day feeding trial, chitosan vitamin E nanocomposite (300 mg/kg) could improve fish resistance against *A. sobria*. These results could be due to the immunostimulating, antioxidant, and antibacterial activity of chitosan NPs in fish; chitosan NPs penetrate the bacterial cell wall and break the cytoplasmic membrane and leakage of its constituents [64]. Other explanations, the antibacterial properties of chitosan could be attributed to electrostatic interaction, contact with the microbial DNA, and metal-chitosan chelation [63], in addition to activating digestive enzymes and inhibiting pathogenic bacteria by activating beneficial ones [65].

## Conclusion

Salt bath at a concentration of 30 ppt caused oxidative stress, lowering the immunity of the experimental Nile tilapia, which became more vulnerable to *S. agalactia* infection. Incorporating CCE-NPs in fish diet pre-salt bath could scavenge the propagated ROS, whereas post-salt bath addition faces low feed intake and stressed body organs that need more time to respond to feed additives. These nanomaterials allow the safe use of high salt concentrations in fish treatment. Unfortunately, the use of dietary-CCE-NPs faces the obstacle of low feed intake and slow onset of their effect at first 24 h post-stress compared to those received supplemented diet pre-stress. Dietary-CCE-NPs could provide higher RLP at 7 and 14 days post-stress against *S. agalactiae* infection.

### Electronic supplementary material

Below is the link to the electronic supplementary material.

Supplementary Material 1

## Data Availability

No datasets were generated or analysed during the current study.
