# Supplementary material for: A blend of chitosan-vitamin C and vitamin E nanoparticles robust the immunosuppressed- status in Nile tilapia treated with salt
Source: BMC Vet Res. 2024 Jul 22;20:331. doi: 10.1186/s12917-024-04180-y (PMC11265070; doi:10.1186/s12917-024-04180-y)
Supplement: Supplementary file 1 — Supplementary Material 1 [file 12917_2024_4180_MOESM1_ESM.docx]

**Supplementary Figures**

A blend of chitosan-vitamin C and vitamin E nanoparticles robust the immunosuppressed- status in Nile tilapia treated with salt

Ahmed H. Sherif, Riad H. Khalil, Talaat S. Talaat, Mahmoud A. Elnagar

**

**

**Figure 1 chitosan vitamin E nano particles and 16.3 to 23.3 nm.**

**

**

**Figure 2 chitosan vitamin C nano particles 11.8 to 14.1 nm.**
